# Humoral Immune Response of SARS-CoV-2 Infection and Anti-SARS-CoV-2 Vaccination in Renal Transplant Recipients

**DOI:** 10.3390/vaccines10030385

**Published:** 2022-03-03

**Authors:** Narayan Prasad, Brijesh Yadav, Mantabya Singh, Sonam Gautam, Dharmendra Bhadauria, Manas Patel, Ravi Kushwaha, Deependra Yadav, Ankita Singh, Monika Yachha, Manas Behera, Anupama Kaul

**Affiliations:** Department of Nephrology and Renal Transplantation, Sanjay Gandhi Postgraduate Institute of Medical Sciences Lucknow, Lucknow 226014, India; brijeshbio146@gmail.com (B.Y.); mantabya321singh@gmail.com (M.S.); sonamgautamlko@gmail.com (S.G.); drdharm1@rediffmail.com (D.B.); drmrpnephro@gmail.com (M.P.); sravikush@gmail.com (R.K.); deependrakgmu@gmail.com (D.Y.); singhanki1202@gmail.com (A.S.); m.yachha@gmail.com (M.Y.); dr.manas.behera@gmail.com (M.B.); anupa@sgpgi.ac.in (A.K.)

**Keywords:** vaccination, anti-SARS-CoV-2 antibody, humoral immunity, Covishield, Covaxin

## Abstract

Vaccination-induced SARS-CoV-2 neutralizing antibodies are required for herd immunity. Vaccine availability and poor vaccine response in renal transplant recipients (RTRs) remain a concern. There is no report on the efficacy of Covaxin and Covishield vaccines in RTRs. We recruited 222 live donors RTRs and analyzed the serum titer of anti-SARS-CoV-2 spike protein antibody by chemiluminescent magnetic microparticle immunoassay. Patients were categorized into three groups: group1 with SARS-CoV-2 infection and no vaccination (*n* = 161); group 2 with only vaccination and no SARS-CoV-2 infection (*n* = 41); and group 3 with both vaccination and SARS-CoV-2 infection (*n* = 20). Overall seroconversion rate was 193/222 (86.9%) with a median titer 1095.20 AU/mL. The median IgG titer value in group 1 was 647.0 AU/mL; group 2 was 1409.0 AU/mL; and group 3 was 1831.30 AU/mL. Covaxin associated seroconversion was observed in 16/19 (84.21%), with a median titer of 1373.90 AU/mL compared to that of Covishield 32/42 (76.19%), whose median titer was 1831.10 AU/mL. The seroconversion rate due to SARS-CoV-2 infection was 145 (90.06%), it was lowest with the vaccination-only group (70.7%), and with both vaccination and SARS-CoV-2 infection group it was highest (95%). In RTRs, SARS-CoV-2 infection and both Covaxin and Covishield vaccination effectively induce a humoral immune response against the SARS-CoV-2 spike protein; however, seroconversion rate was lower and the antibody titer was higher with vaccine than infection.

## 1. Introduction

The induction of SARS-CoV-2 neutralizing antibodies, its neutralizing activities, and duration of persistence remain disputed challenges for success of the vaccination program across the globe [1,2,3]. The vaccination-associated immune response is further limited in immunocompromised patients, such as those with renal transplant recipients (RTR). A study by Arevalo et al. showed that kidney transplant and dialysis patients respond poorly to the vaccination in terms of T and B cell activation and antibody response against mRNA vaccines BNT162b2 (Bionet/Pfizer) [4]. The mRNA-based vaccination in western renal transplant patients shows a poor seroconversion rate after 28 days of the second dose of immunization [1,4,5]. The availability of the mRNA-based vaccines is limited to developed countries, and there is a scarcity of vaccines to cover the world’s entire population. Adenovirus vector-based ChAdOx1-nCOV (Covishield™) and inactivated whole virus-based BBV-152 (Covaxin™) vaccines are available in India. These may cover a large population at a lower cost. However, the efficacy of these vaccines in inducing anti-SARS-CoV-2 humoral response was not studied in immunocompromised RTRs, a group more vulnerable to acquiring SARS-CoV-2 infection, developing severe COVID-19, and mortality compared to that of the general population [6]. RTRs may not achieve adequate immune response and seroconversion after vaccination, as evident from the currently available data from the most efficacious mRNA anti-SARS-CoV-2 vaccine in the general population [1,7]. A few studies also reported adverse events of vaccination leading to sporadic allograft rejection [1,8,9]. The study shows that RTRs with prior SARS-CoV-2 infection show a better immune response after vaccination but are less efficient than hemodialysis patients with the previous COVID-19, suggesting inadequate immune response in RTR [10]. The poor humoral response in an immunocompromised population is largely known. The immunosuppressive agents that prevent organ rejection result in activation of latent viruses, thereby causing virus-related diseases such as progressive multifocal leukoencephalopathy caused by polyomavirus JC, and BK virus nephropathy caused by latent BK polyomavirus activation [11,12]. The physiological immune modulation during pregnancy was also reported to increase Merkel cell polyomavirus activity [13]. The activation of viruses induces proinflammatory cytokines secretion and hamper IgG seroconversion [13].

In healthy healthcare workers, a study showed that both ChAdOx1-nCOV (Covishield™) and BBV-152 (Covaxin™) induce a robust humoral response in the Indian population [2]. However, a few people still contracted SARS-CoV-2 infection even after receiving complete vaccination doses, albeit with lower hospitalization and reduced mortality rates [14]. In India, the majority of people are being vaccinated with either Covishield™ (a replication-deficient Adenovirus vector encoding SARS-CoV-2 spike protein) of Oxford–AstraZeneca or Covaxin™ (an inactivated whole virus) of Bharat biotech and Indian council for medical research (ICMR). The efficiency of these vaccines in RTRs is still unknown. The antibody response after vaccination in those who already had SARS-CoV-2 infection and those who do not have an infection are unknown. The vaccine response in terms of quantitative neutralizing antibody titer in asymptomatic infection and those with and without symptomatic infection may vary; however, it remains undiscovered. In the current study, we aimed to measure the humoral response with IgG seroconversion rate with anti-SARS-CoV-2 Spike protein in RTRs vaccinated with either Covishield™ or Covaxin™. 

## 2. Materials and Methods

### 2.1. Patient Recruitments

A total of 222 consecutive RTRs who attended the outpatient transplant clinic during the study period from 1 June to 21 July 2021 were included in the study. Every patient was negative with SARS-CoV-2 reverse transcriptase-polymerase chain reaction (RT-PCR) testing at the time of inclusion in the study. Only RT-PCR-negative patients were allowed for the outpatient clinic visit. The institute’s ethics committee approved the study. The informed consent form was obtained from each patient for inclusion in the study. The study was conducted in adherence to the declaration of Istanbul and the declaration of Helsinki. 

A detailed history of SARS-CoV-2 infection, confirmed with RT-PCR in the last six months, as well as history of hospitalization and vaccination were obtained from each patient. The details of transplantation were obtained from the electronic record of the institute. Patients with a single vaccine dose were excluded from the study. Patients who already had a second vaccination dose at least 14 days before the study visit, at an interval of two to three months between the two doses of vaccines, were included in the vaccination group. 

Patients were categorized into three groups based on their vaccination history and previous SARS-CoV-2 infection for the analysis purpose. Group 1 patients had previous SARS-CoV-2 infection and no vaccination (*n* = 161). Group 2 patients had both doses of vaccination only, without asymptomatic or symptomatic SARS-CoV-2 infection. This patient group tested themselves for SARS-CoV-2 antibodies before vaccination and were included in this group only when they had a negative report for antibodies by any method (*n* = 41). Group 3 patients had both vaccination doses and SARS-CoV-2 infection (*n* = 20). The vaccination of each individual was confirmed with the vaccination certificate issued by the Ministry of Health and Family Welfare, Government of India (MOHFW, GOI). 

### 2.2. Anti-SARS-CoV-2 Spike Protein IgG Titer Measurement

A blood sample of 5 mL in a plain vial containing clot activating factor was collected from each patient irrespective of their prior history of SARS-CoV-2 infection and vaccination. Blood was centrifuged at 2000RPM for 10 min, and serum was separated and stored immediately in −80 °C till the analysis. Immunoglobulin-G (IgG) titer against SARS-CoV-2 spike protein antigen was measured using the chemiluminescent magnetic microparticle Immunoassay (CMIA)-based analyzer as per the manufacturer’s instruction. In brief, SARS-CoV-2 antigen-coated paramagnetic microparticles were incubated with serum in assay diluent, followed by washing and incubation with acridinium-labeled antihuman IgG conjugate. Following this, washing was performed, and pretrigger hydrogen peroxide solution and triggered sodium hydroxide solution were added. Pretriggered solution split the acridinium dye off from IgG, and triggered solution oxidized the acridinium dye, leading to a chemiluminescent reaction, which was measured as a relative light unit (RLU). RLU was directly proportional to the amount of anti-SARS-CoV-2 IgG present in samples. Sample RLU values were normalized with the calibrator RLU [15]. A reference cutoff value ≥ 50 AU/mL was considered positive for anti-SARS-CoV-2 spike protein IgG and defined as seroconversion as per manufacturer’s instruction. The maximum detection limit of the kit was 40,000 AU/mL. In the in vitro assay, a IgG titer cutoff value ≥50 AU/mL was reported to neutralize the SARS-CoV-2 virus infection in VERO cell lines [16].

### 2.3. Statistical Analysis

Statistical analysis was performed with the SPSS software version 20 (IBM, corporation, Armonk, NY, USA). Variables were tested for normality distribution with Shapiro–Wilk test. One-way analysis of variance (ANOVA) was used to calculate mean and standard deviation for continuous variables in multiple groups for parametric variables, and Kruskal–Wallis test was applied for nonparametric variables; values were expressed as a median. Median and interquartile range (IQR) were calculated for the IgG antibody titer level. A descriptive and inferential analysis was performed to interpret the result. Categorical variables were analyzed using the Chi-square test or Fischer exact test as per application required, and categorical values were expressed in percentages. Graphs were plotted using GraphPad Prism version 8 for Windows, GraphPad Software, La Jolla, CA, USA.

## 3. Results

### 3.1. Demographic and Clinical Characteristics of Patients

Patients’ demographic and baseline clinical characteristics were similar amongst the groups, except the age, post-transplant interval, BMI, and induction regimen, as shown in Table 1.

### 3.2. Seroconversion Rate in Different Groups

The seroconversion rate in group 1 patients was observed in 90.0% (*n* = 145/161) of patients; in group 2, it was 70.73% (*n* = 29/41), and in group 3, it was 95% (*n* = 19/20). The seroconversion of 90.06 % in group 1 indicates that most patients had an asymptomatic or symptomatic infection and developed anti-SARS-CoV-2 spike protein antibodies. The seroconversion was lowest (70.73%) in patients only with vaccination and the highest 95% in those with both vaccination and SARS-CoV-2 infection.

### 3.3. Anti-SARS-CoV-2 Spike Protein IgG Antibody Titer in Different Groups

**Figure 1:**
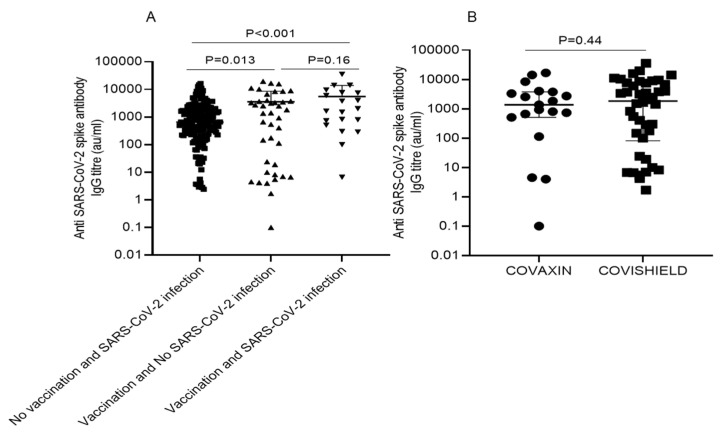
(**A**) Showing differences in anti-SARS-CoV-2 antibody titer between three groups; (**B**) anti-SARS-CoV-2 antibody titer between Covaxin and Covishield.

The anti-SARS-CoV-2 spike protein IgG antibody titer in all three groups is shown in Table 2. Out of 222 patients, 86.9% (193/222) developed anti-SARS-CoV-2 spike protein IgG antibodies. The median titer was 1095.20AU/mL, (IQR: 384.85–2823.15). The median IgG titer value of group 1 patients (*n* = 161) was 647.0 AU/mL (IQR: 229.1–1819); in group 2 patients (*n* = 41), it was 1409.0AU/mL (IQR: 14.50–3882.55); and in group 3 patients (*n* = 20), it was 1831.30AU/mL (IQR: 565.25–7644.65). The vaccination group had a higher antibody titer than nonvaccinated SARS-CoV-2 infection. The antibody titer was lowest in group1 with SARS-CoV-2 infection, moderate in group 2 who had vaccine only, and highest in group 3, those who had both vaccination and infection in the past (Table 2, Figure 1A).

### 3.4. Covaxin™ Versus Covishield™ Seroconversion Rate

A total of 27.4% (*n* = 61/222) patients, 41 in group 2 and 20 in group 3, received the vaccines, either Covaxin™ or Covishield™. Out of these, 78.68% (*n* = 48/61) patients developed anti-SARS-CoV-2 spike IgG antibodies, and 21.31% (*n* = 13/61) did not develop antibodies (*p* = 0.002) Table 3.

A total of 42 patients received Covishield, and 19 received Covaxin. Out of 42 patients with Covishield vaccination, 76.19% (32/42) had seroconversion, and 23.80% (10/42) did not have seroconversion (*p* = 0.003). Out of 19 patients with Covaxin, 84.21% (16/19) had seroconversion, and 15.7% (3/19) did not have seroconversion (*p* = 0.016). The seroconversion rate between Covaxin and Covishield was statistically similar (*p* = 0.44). The median titer of anti-SARS-CoV-2 spike IgG antibody in the Covaxin group was 1373.90 (IQR: 507.80–3765.10) AU/mL, and for those vaccinated with Covishield, it was 1831.10 (81.26–7522.56) AU/mL; however, the median titer was lowest in those who had infection with and without symptoms: 647.80 (229.15–1819.65) AU/mL. (Table 3, Figure 1B).

### 3.5. Clinical Variables Associated with Seroconversion

The seroconversion rate in patients of the 18–45 years age group was 90.20% (*n* = 130/144), in the 46–60 years age group it was 82.35% (*n* = 56/68), and in the >60 years group it was 70% (*n* = 7/10). The seroconversion decreased with increasing age; however, the seroconversion rate was statistically not different. There was no impact on blood groups, immunosuppression regimen, post-transplant interval, body mass index, or serum creatinine on seroconversion. Similarly, patients with AB^+ve^ blood group had a seroconversion rate of 93.10% (*n* = 27/29), the O^+ve^ blood group had 89.06% (*n* = 57/64), A^+ve^ had 87.03% (*n* = 47/54), and B^+ve^ had 82.66% (62/75). Those patients who had serum creatinine value <1.4 mg/dL had seroconversion in 87.59% (*n* = 120/137) of patients, compared to patients who had serum creatinine value of >1.4 mg/dL in 85.88% (*n* = 73/85). The seroconversion tended to be poor, with a declining estimated glomerular filtration rate (Table 3). The major side effects observed with both the vaccines were fever, myalgias, headache, body ache, and giddiness, and the differences between Covaxin and Covishield were not significant.

## 4. Discussion

A massive vaccination program against the SARS-CoV-2 virus was launched globally to develop herd immunity in the community to prevent virus transmission [17]. The RTRs are a particular group of the immunocompromised population with a higher risk of infection and mortality and a relatively lower vaccine response because of ongoing immunosuppression. Most patients obtain triple immunosuppression, calcineurin inhibitors, mycophenolate mofetil (MMF), and steroids, which block the cellular and humoral immune, resulting in a poor seroconversion rate [1]. A significant proportion develops re-infection despite two doses of vaccination [14], and even after 3rd dose [18,19]. Only 49–64% of organ transplant recipient patients develop anti–SARS-CoV-2 antibodies after vaccination with mRNA-based BNT162b2 (Pfizer–BioNTech) [19,20]; meanwhile, the seroconversion rate was higher with mRNA-based vaccine in the general population. In a cohort study of 205 kidney transplant patients, only 98 (47.80%) patients develop IgG seroconversion after the second dose.

Similarly, a 44% seroconversion was observed after ChAdOx1 (Covishield) vaccination in UK-based renal transplant recipients cohort [20], and a lower IgG titer was observed in the German solid organ transplant cohort [21]. A lower seroconversion rate is expected in RTRs than that of the general population. Patients on calcineurin inhibitors, MMF, and steroids had poor seroconversion rates [1,7]. The mRNA-based vaccines are costlier, and availability is limited to cover the entire vaccine-eligible population. In the present study, we observed that the seroconversion rate with Covishield and Covaxin is comparable, or relatively higher, to that of the mRNA-based vaccines. Infection with SARS-CoV-2 usually triggers the generation of high titers of protective neutralizing anti-spike IgGs in RTRs, similar to our observations [22]. A study also revealed that the main predictor of successful seroconversion after vaccination is a previous history of COVID-19 in RTRs. A single dose of mRNA vaccine leads to a 100% seroconversion rate in such RTRs and antibody levels that are comparable to those observed in healthy volunteers [23]. The seroconversion depends on the vaccine immunogenicity, which also depends on adjuvants, mode of delivery, and antigen doses. The composition of vector-based Covishield vaccine includes inactivated adenovirus with spike protein of SARS-CoV-2, and Covaxin includes inactivated coronavirus, along with multiple adjuvant lipids, salts, sugars, and acidic substances. In general, the vector-based vaccine and inactivated vaccine approaches were known for decades. The mRNA-based vaccine is a novel method, and the optimization of any vaccine immunogenicity in RTRs is yet to be established [24]. The observed difference in antibody response may be because of the differing antigenicity of vaccine. It has higher seroconversion with infection; however, antibody titers fade with duration [25]. The high seroconversion rate and titer of 95% in group 3 with both vaccination and infection in our cohort is expected. The high seroconversion rate of 70% in the exclusively vaccinated group 2 patients is higher than that of the m-RNA-based vaccine in RTR, which may be because of differing immunogenic potential of spike protein in vector-based Covishield and inactivated virus in Covaxin in RTRs. The other reason could be that some of the group 2 patients also had asymptomatic infection and titer faded with time before vaccination, so they were therefore not detectable at the time of vaccination.

To date, as per World Health Organization (WHO) data, SARS-CoV-2 caused 42.7 million cases and 0.51 million deaths due to COVID-19 in India [26,27]. Uttar Pradesh, the most densely populated state of India, which is also where the institute is located, witnessed a community spread that was hard hitting with SARS-CoV-2 infection. It also affected RTRs symptomatically and asymptomatically, leading to undetected seroconversion in many patients [28]. In our cohort, the overall seroconversion rate was 86.93% (193/222). In the majority of patients, 90.06% (145/222) acquired SARS-CoV-2 infection and developed anti-SARS-CoV-2 antibodies. We noticed that although SARS-CoV-2 infection induced a humoral immune response and seroconversion, our cohort had a lower anti-spike protein antibody titer. A few studies reported that a SARS-CoV-2 viral infection induces a humoral reaction equivalent to a single dose of vaccination [29,30]. Although we did not study the single-dose vaccine response, we observed that the antibody titer was highest in those who have confirmed SARS-CoV-2 infection and vaccination. The vaccination in such patients can boost the sufficient antibody response. It may also address the issue of fading immune response with duration. Even subjects who developed enough antibodies after vaccination lose it over the short span of a few months, and they probably also require a higher titer of antibody for the complete neutralization of viruses. At present, there is no defined threshold of antibody titer to protect from repeated infection [31]. However, invitro assay showed that >50 au/mL is sufficient in preventing viral infection to VERO cell line [17]. It also appears that increasing vaccine immunogenicity might be a safer approach to improving vaccine efficacy and reducing severity, hospitalization, and mortality in such vulnerable patients, rather than reducing immunosuppression during treatment, which may aggravate rejection in RTRs [32]. We strongly recommend to prioritize the vaccination of this vulnerable population.

## 5. Conclusions

The majority of renal transplant recipients (RTRs) had anti-SARS-CoV-2 spike protein IgG seroconversion. It may either be due to true seroconversion due to vaccination or may be due to SARS-CoV-2 infection. However, vaccination significantly improves the SARS-CoV-2 neutralizing antibody titer. Both Covaxin and Covishield vaccines had a better seroconversion rate in the Indian transplant patients. Vaccination should be promoted in RTRs, a vulnerable group of high COVID-19-related mortality. However, this is a preliminary report on the vaccination and safety of Covishield and Covaxin vaccines in RTRs in India. A more extensive study is required to confirm the findings in a multicenter-based study.

## Figures and Tables

**Table 1:** Demographic and clinical characteristics of patients.

Characteristics		Total	No Vaccination and SARS-CoV-2 Infection (*n* = 161)	Vaccination and No SARS-CoV-2 Infection (*n* = 41)	Vaccination and SARS-CoV-2 Infection (*n* = 20)	*p* Value
Age (Years)		41.23 ± 10.20	39.28 ± 9.69	45.95 ± 8.22	47.30 ± 10.61	<0.001 *
Gender	M	195	142	35	18	0.83 ***
	F	27	19	6	2	
Post-transplant interval in Month (range)		74.33 (2.0–276)	65.0 (2.0–216.0)	96 (20.0–240.0)	94.10 (12.0–276.0)	0.04 **
BMI(Kg/M^2^)		24.28 ± 5.21	23.76 ± 5.20	25.32 ± 4.51	26.36 ± 6.04	0.04 *
Hemoglobin (g/dL)		12.98 ± 1.82	13.05 ± 1.88	13.03 ± 1.45	12.28 ± 1.96	0.19 *
Baseline serum creatinine (mg/dL)		1.01 ± 0.37	1.02 ± 0.37	0.95 ± 0.33	1.05 ± 0.40	0.48 *
Serum creatinine (mg/dL)		1.41 ± 0.71	1.46 ± 0.79	1.20 ± 0.34	1.40 ± 0.49	0.11 *
TLC (×10^3^/µL)		8.32 ± 2.48	8.25 ± 2.61	8.57 ± 1.86	8.41 ± 2.53	0.75 *
BUN (mg/dL)		17.85 (5.80–59.80)	17.80 (5.80–59.80)	16.0(7.16–35.0)	20.20(6.60–48.10)	0.32 **
eGFR (mL/min)		70.13 ± 33.31	69.33 ± 36.28	74.56 ± 22.01	67.45 ± 27.23	0.62 *
Tacrolimus level (µg/L)		5.20 (2.0–16.90)	5.40 (2.10–16.90)	4.85 (2.40–11.80)	4.80 (2.0–16.60)	0.48 **
Systolic BP (mmhg)		130.88 ± 14.47	130.55 ± 14.86	133.27 ± 13.68	128.65 ± 12.82	0.43 *
Diastolic BP (mmhg)		80.73 ± 9.90	80.82 ± 9.95	80.39 ± 10.27	80.75 ± 9.08	0.97 *
Vaccine Brand recipients	Covishiled	42	0	26	16	<0.001 ***
	Covaxin	19	0	15	4	
Patient blood group	A+	44	44	4	6	0.13 ***
	B+	75	81	20	4	
	O+	64	47	10	7	
	AB+	29	19	7	3	
Induction regimen	None	104	66	25	13	0.028 ***
	Basiliximab	93	78	10	5	
	ATG	25	17	6	2	
MMF+ Steroid+	Tacrolimus	212	156	38	18	0.23 ***
	Cyclosporin	10	5	3	2	

Foot Note: * ANOVA, analysis of variance test, ** Kruskal–Wallis test, *** Chi square test. BMI, body mass index; BUN, blood urea nitrogen; BP, blood pressure.

**Table 2:** Anti-SARS-CoV-2 spike protein antibody titer and seroconversion ^$^.

Sr. No	No Vaccination and SARS-CoV-2 Infection (*n* = 161)		Vaccination and No SARS-CoV-2 Infection (*n* = 41)		Vaccination and SARS-CoV-2 Infection (*n* = 20)		*p* Value
Median and IQR of antibody titer (AU/mL)	647.0 (IQR: 229.1–1819.65) ^a^		1409.0 (IQR: 14.50–3882.55) ^b^		1831.30 (IQR: 565.25–7644.65) ^c^		a vs. b, *p* = 0.013 a vs. c, *p* < 0.001 b vs. c, *p* = 0.16
Over all anti-SARS-CoV-2 spike protein antibody seroconversion	Yes (*n* = 145; 90.06%)	No (*n* = 16; 9.93%)	Yes (*n* = 29; 70.73%)	No (*n* = 12; 29.26%)	Yes (*n* = 19; 95.0%)	No (*n* = 1; 5%)	0.002

^$^ Indicates seroconversion in patient who had anti-SARS-CoV-2 Spike protein antibody titer >50 AU/mL. ^a^- No Vaccination and SARS-CoV-2 Infection; ^b^- Vaccination and No SARS-CoV-2 Infection; ^c^- Vaccination and SARS-CoV-2 Infection.

**Table 3:** Different factors influencing seroconversion rate in allograft recipient patients.

Characteristics		Seroconversion		
		Yes	No	*p* Value
Post-transplant interval (months) associated seroconversion	2–60	73 (87.95%)	10 (12.04%)	0.71
	60–120	72 (84.7%)	13 (15.3%)	
	120–180	31 (86.1%)	5 (13.8%)	
	>180	17 (94.4%)	1 (5.5%)	
BMI (kg/m^2^) associated seroconversion	<18.4	*n* = 37 (90.2%)	*n* = 4 (9.7%)	0.23
	18.4–24.99	*n* = 72 (82.75%)	*n* = 15 (17.24%)	
	25.29–29.99	*n* = 62 (92.5%)	*n* = 5 (7.4%)	
	>30	*n* = 22 (81.5%)	*n* = 5 (18.51%)	
Age-wise (Year) seroconversion ^$^	18–45	*n* = 130 (90.27%)	*n* = 14 (9.72%)	0.074
	46–60	*n*= 56 (82.35%)	*n* = 12 (17.46%)	
	>60	*n* = 7 (70%)	*n* = 3 (30%)	
Vaccine brand associated seroconversion	Covishield™	*n*= 32 (76.19%)	*n* = 10 (23.80%)	0.003
		Titer: 1831.10 (81.26–7522.56) AU/mL		
	Covaxin™	*n* = 16 (84.21%)	*n* = 3 (15.78%)	0.016
		Titer: 1373.90 (IQR: 507.80–3765.10) AU/mL		
Blood group associated seroconversion	A+	*n* = 47 (87.03%)	*n* = 7 (12.96%)	0.488
	B+	*n* = 62 (82.66%)	*n* = 13 (17.33%)	
	AB+	*n* = 27 (93.10%)	*n* = 2 (6.89%)	
	O+	*n* = 57 (89.06%)	*n* = 7 (10.93%)	
Immunosuppression associated seroconversion	Tacrolimus + MMF + Steroid	*n* = 184 (86.79%)	*n* = 28 (13.20%)	1.00
	Cyclosporin + MMF + Steroid	*n* = 9 (90%)	*n* = 1 (10%)	
Serum creatinine	<1.4	120 (87.59%)	17 (12.40%)	0.83
	>1.4	73 (85.88%)	12 (14.11%)	

^$^ As per ICMR guideline of vaccination.

## Data Availability

The data supporting this study’s findings are available from the corresponding author upon reasonable request.

## Abbreviations

SARS-CoV-2-, severe acute respiratory syndrome coronavirus 2; BMI, body mass index; eGFR, estimated glomerular filtration rate; TLC, total leucocyte count.
